# The effect of selective serotonin and norepinephrine reuptake inhibitors on clinical outcome of COVID‐19 patients: A systematic review and meta‐analysis

**DOI:** 10.1002/hsr2.892

**Published:** 2022-10-17

**Authors:** Dena Firouzabadi, Fatemeh Kheshti, Saeed Abdollahifard, Erfan Taherifard, Mohammad Reza Kheshti

**Affiliations:** ^1^ Department of Clinical Pharmacy, School of Pharmacy Shiraz University of Medical Sciences Shiraz Iran; ^2^ Shahid Faghihi Hospital Shiraz University of Medical Sciences Shiraz Iran; ^3^ Student Research Committee Shiraz University of Medical Sciences Shiraz Iran; ^4^ Research Center for Neuromodulation and Pain Shiraz Iran; ^5^ MPH department Shiraz University of Medical Science Shiraz Iran

**Keywords:** COVID‐19, selective serotonin reuptake inhibitors, serotonin‐norepinephrine reuptake Inhibitor, SNRI, SSRI

## Abstract

**Background and Aim:**

Due to the high social and economic burden and also mortality and morbidity caused by coronavirus disease 2019 (COVID‐19) in the past few years, researchers have aimed at finding solutions to suppressing the severity of infection. Recently, selective serotonin and serotonin‐norepinephrine reuptake inhibitors (SSRI/SNRI) have been investigated as an adjuvant treatment for COVID‐19. The aim of the current study was to investigate the impact of SSRI/SNRIs on outcomes of COVID‐19 patients.

**Methods:**

In this systematic review and meta‐analysis, a comprehensive search strategy consisting of relevant words was performed by two researchers in PubMed, Scopus and EMBASE libraries. Studies reporting the effect of SSRI and/or SNRI use in COVID‐19 patients' outcome were included. Hospitalization, mortality, hospitalization event, and length of hospital stay were considered as main outcomes of this study. Analysis was carried out using Comprehensive Meta‐Analysis (CMA‐version 2) and final data were reported as odds ratio (OR) and 95% confidence interval (CI).

**Results:**

Our search led to the final selection of 9 articles including 15,287 patients. The effect of fluvoxamine, fluoxetine, and the overall effect of SSRI/SNRI use on mortality of COVID‐19 patients were investigated in 3, 2, and 7 articles, respectively. The results of our analyses showed that these medications could significantly decrease mortality of COVID‐19 patients (OR and 95% [CI]: 0.595 [0.467–0.758], 0.620 [0.469–0.821], and 0.596 [0.437–0.813]). The effect of SSRI/SNRIs on hospitalization events of COVID‐19 patients was not significant (OR: 0.240% and 95% CI: 0.041–1.4). Also, length of hospital stay was longer in patients who administrated SSRIs.

**Conclusion:**

According to this study's results, SSRI/SNRIs may be effective in reducing mortality of COVID‐19 patients, suggesting the superiority of fluvoxamine to fluoxetine. The safety profile and affordable cost of SSRI/SNRIs for a short‐term use may be other reasons to propose them as beneficial medications in preventing mortality in COVID‐19.

## 1 INTRODUCTION

From the beginning of the coronavirus disease 2019 (COVID‐19) pandemic in early 2020, a global endeavor has been started to find the best treatment, and an outstanding progress has been accomplished to minimize the transmission and complications of this infectious disease.^1^ Although the basic mechanism and origin of this disease are still under investigation, male sex, obesity, higher age, immunosuppression, and comorbidities such as diabetes and chronic kidney disease are considered risk factors that are related to the severity of COVID‐19.^2^, ^3^ Transmission of COVID‐19 could occur by droplets spreading through human‐to‐human contacts or touching contaminated surfaces, and the infection can be considered as an airborne contagion.^4^ It has been shown in a short‐time study that climate does not influence outbreaks of COVID‐19.^5^, ^6^ The transmission dynamics of COVID‐19 was found to be related to air pollution as well.^7^ Remdesivir, favipiravir, hydroxychloroquine, azithromycin, and corticosteroids were all a part of a world‐wide effort to treat this disease.^8^, ^9^, ^10^, ^11^, ^12^ For the first time, a randomized clinical trial was performed on fluvoxamine, as a selective serotonin reuptake inhibitor (SSRI), to determine the effect of this drug on the clinical deterioration of symptomatic COVID‐19 patients and showed a promising impact on preventing the progress of the disease.^13^ After this study, other SSRIs and specifically fluoxetine were studied for their effect on the course of COVID‐19 infection.^14^, ^15^ The exact mechanism of SSRI/serotonin‐norepinephrine reuptake inhibitor (SNRI) on coronavirus is not well known but there are some theories and explanations justifying its probable efficacy.^16^ SSRIs are a part of functional inhibitors of acid sphingomyelinase and Coronavirus has been proven to use acid sphingomyelinase to enter the cells and as a result, these drugs may inhibit the entrance of the virus into the respiratory and other human cells.^17^, ^18^ This mechanism leads to decrease in cytokine and interleukin release,^19^, ^20^ conveying a positive effect on interference with host cells.^21^ Showing anti‐inflammatory effects^22^ decrease in the aggregation of platelets and modulation of the degranulation of mast cells^16^, ^23^, ^24^ are all of the many mechanisms described for their protective effects. Although many treatments strategies and vaccines have been developed and introduced during the past 2 years that have reduced complications and most importantly mortality rates, there still exists major concerns regarding the pathogenicity of probable new variants and future outbreaks due to the inefficient available data regarding the pathophysiology of the disease and ways to best control its complications.^25^ A remarkable decrease in mortality and improvement of patients' clinical outcomes have been reported with SSRI/SNRIs in COVID‐19 infection but the actual effect of these drugs remains questionable.^26^ Considering that antidepressants such as SSRI/SNRIs may be used widely in the general population with their limited serious adverse effects and their manageable cost for short‐term use, the benefits or drawbacks of these medications may be worthy of attention for use in COVID‐19 infected patients.

In this systematic review and meta‐analysis, we aimed to assess the impact of SSRI/SNRI use on mortality, hospitalization, and length of hospital stay in COVID‐19 patients. Also, we are going to review eligible studies and investigate the accurate findings of each study.

## 2 METHODS

This study was carried out according to Preferred Reporting Items for Systematic reviews and Meta‐Analyses (PRISMA) guideline.^27^ A systematic search of online databases was performed to detect published articles investigating the effect of SSRI and SNRI drugs on confirmed COVID‐19 patients, repurposing their use, if effective, in COVID‐19 infection.

### 2.1 Eligibility criteria

Studies that revised the effect of SSRI and/or SNRIs as repurposing drugs on PCR confirmed COVID‐19 patients (hospitalized and nonhospitalized) and reported the drugs' effect on the outcome of the patients (mortality, hospitalization, and length of hospital stay), were found and selected to be included in this study. Selected study designs were randomized clinical trials, cohorts, case‐control, and cross‐sectional studies. We excluded the studies which were in vitro, cellular, molecular, hypothesis, non‐English, conference papers, case series, case reports, reviews, editorials, unpublished or ongoing studies, or abstracts.

### 2.2 Information source

The search process was performed on April 2022. Two separate authors have searched three electronic databases, including MEDLINE/PubMed, EMBASE, and Scopus, at the same time. For covering the possible missed studies, references of the final selected articles have also been evaluated for relevance to this study.

### 2.3 Search strategy

We prepared a synonym table for our keywords. The selected synonyms for COVID‐19 (identified by keywords and National Library of Medicine's MESH) were *“COVID 19, SARS‐CoV‐2 Infection, SARS CoV 2 Infection, 2019 Novel Coronavirus Disease, 2019 Novel Coronavirus Infection, 2019‐nCoV Disease, 2019 nCoV Disease, COVID‐19 Virus Infection, Coronavirus Disease 2019, Coronavirus Disease‐19, Coronavirus Disease 19, Severe Acute Respiratory Syndrome Coronavirus 2 Infection, SARS Coronavirus 2 Infection, COVID‐19 Virus Disease, COVID 19 Virus Disease, 2019‐nCoV Infection, 2019 nCoV Infection, COVID19, COVID‐19 Pandemic, COVID 19 Pandemic”* that were combined together by using “OR” and then combined by using “AND” with the relevant words to SSRI/SNRIs which were *“SSRI, Serotonin Uptake Inhibitors, 5‐Hydroxytryptamine Uptake Inhibitor, Serotonin Reuptake Inhibitor, Serotonin Uptake Inhibitor, 5‐HT Uptake Inhibitor, Selective Serotonin Reuptake Inhibitor, Citalopram, Escitalopram, Fluoxetine, Paroxetine, Sertraline, SSRI, SNRI, Serotonin and Norepinephrine Reuptake Inhibitors, Serotonin and Noradrenaline Uptake Inhibitors, Serotonin and Norepinephrine Uptake Inhibitors, Desvenlafaxine, duloxetine, levomilnacipran, venlafaxine, SNRI”* to make the final search strategy. The used filters in PubMed and EMBASE were the Title‐abstract filter, and for Scopus, we used the Title‐abstract‐keywords filter. There were no limits in the search process. The search details in each database are provided in the Supporting Information Materials.

### 2.4 Selection and data collection process

**Figure 1:**
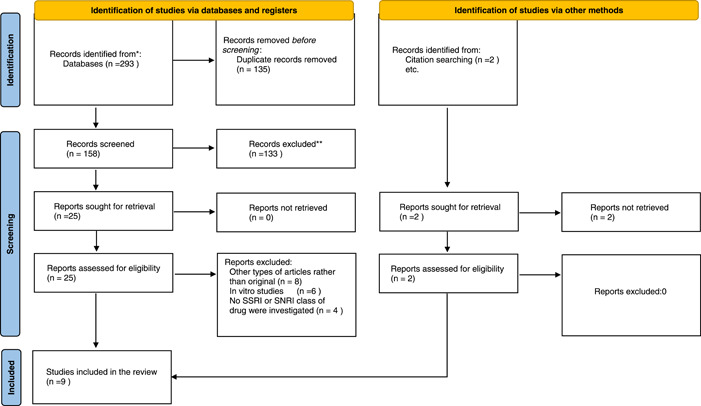
PRISMA flowchart of included studies for determining the effect of SSRI/SNRI on clinical outcomes of COVID‐19 patients. COVID‐19, coronavirus disease 2019; PRISMA, Preferred Reporting Items for Systematic reviews and Meta‐Analyses; SNRI, serotonin‐norepinephrine reuptake inhibitor; SSRI, selective serotonin reuptake inhibitor

The selection and data collection process were performed manually, not using any filter or automatic tools, by two separate authors working parallel to each other. In the cases of noticing any conflicts between researchers, consultation with the third author was mandatory. All the search results from each database were exported to an endnote library, then duplications were removed. In the first step of the selection process, two authors have screened the articles' titles and abstracts, selected articles from this step entered the second step which was reading the full text of the articles and finding the matched articles with the deliberated eligibility criteria. The included articles after step 2, were discoursed in the group again, the decision on which outcomes to be extracted was made and the accuracy of extrapolated data was checked in between all authors. Figure 1 reflects the PRISMA flowchart of selection and data collection process.

### 2.5 Data items

We attempted to extract the desirable demographic data and clinical outcomes from the selected studies. Study design, number of patients (case/control based on their design), study population, intervention/placebo, or exposure were our extracted demographic data. Our main outcomes were mortality, hospitalization event, and length of hospital stay.

### 2.6 Risk of bias assessment

The risk of bias and quality of each article were weighed by two separate authors, using National Institutes of Health (NIH) tools for evaluating the quality of studies.^28^ The mentioned tool consists of questions assessing different parts of the studies such as randomization, allocation, blinding, selection, and selective reporting bias and validation for randomized clinical trials and defining population and exposure for observational or cohort studies. For each question, three options of “Yes” for being according to that index, “No” for not properly considered, and “N.A” or not applicable for not reported are considered.

### 2.7 Effect measures and synthesis methods

Comprehensive meta‐analysis version 2 was used to determine the effect size of this study. Five different analyses have been performed using random model analysis. First, the effect of SSRI/SNRIs on the mortality of COVID‐19 patients. Second, the effect of fluvoxamine on mortality of COVID‐19 patients. Third, the effect of fluoxetine on mortality of COVID‐19 patients. Fourth, the effect of Fluoxetine and/or Fluvoxamine on mortality of COVID‐19 patients. At last, the effect of SSRIs on hospitalization events of COVID‐19 patients. Final data was reported in a forest plot and as an odds ratio (OR). Heterogeneity of data was explored by using *I* ^2^ index, a *p* value less than 0.05 was considered significant and all statistical tests were two‐sided. For determining the risk of publication bias, Eager's test was used. The sensitivity of results to an article has been assessed by ignoring each article at a time and then re‐analyzing the rest of the articles.

## 3 RESULTS

Our search resulted in 293 articles which after eliminating duplications 158 articles remained for the two‐step screening process. Overall, 15,287 patients were included in this study. The screening process resulted in a remaining number of 7 articles that were selected for this study and further evaluated. After assessing the references of selected articles, another 2 articles were added manually, and at last 9 articles were included in our systematic review. PRISMA flowchart broadly explains the whole study selection process (Figure 1).

Among our selected studies for this systematic review, 2 studies were double‐blind randomized controlled trials (RCTs). One of them used 100 mg of Fluvoxamine twice a day for 10 days in the intervention group (741 cases) and standard COVID‐19 therapy for the control group (756 placeboes), in high‐risk Brazilian adults with outpatient symptomatic COVID‐19. The two groups' hospital admission events, mortality, length of hospital stay, mechanical ventilation (MV) and its duration, emergency visits, and viral clearance were evaluated in this study.^29^ The other RCT, used Fluvoxamine 100 mg three times a day for 15 days as their intervention (80 cases) versus standard therapy in the control group (72 placeboes), in COVID‐19 nonhospitalized patients and compared different adverse events, clinical deterioration, mortality, and the 30‐day posttrial observation events in the two mentioned groups.^13^ It should be mentioned that in both treatment and control arms of the two studies, patients received standard of care therapy.

Another two studies selected for analysis, were prospective cohorts: one of them was a trial in COVID‐19 intensive care unit (ICU) admitted patients, using Fluvoxamine 100 mg three times a day in 51 patients versus standard therapy in their matched control group (51 control), studying the effect of fluvoxamine on mortality, length of hospital and ICU stay, and days on MV.^30^ The other was a study using Fluvoxamine 50–100 mg loading dose then 50 mg twice daily for 14 days as an early treatment of PCR‐positive COVID‐19 outpatient subjects (65 patients) and evaluated intervention versus no treatment (48 patients) by gauging both groups' mortality and hospital admission.^31^

The design of the three articles was a retrospective cohort. One of them was a retrospective case‐control in which fluoxetine with the dose of 20 mg once daily was given to 110 hospitalized cases with moderate to severe COVID‐19 and their outcome (mortality, C‐reactive protein, Lactate dehydrogenase, D‐dimer) versus the outcome of those with standard therapy (159 matched patients) was assessed.^14^ The second study evaluated the effect of antidepressant therapy (345 receiving any antidepressant including 195 SSRI, 59 SNRI) on the outcome of hospitalized COVID‐19 patients (primary endpoint was intubation or death) compared with other similar patients receiving no antidepressants (6885 not receiving any antidepressants at all).^15^ Finally, the last retrospective cohort study evaluated the mortality rate of 3401 COVID‐19‐19 patients receiving SSRI therapy (470 fluoxetine, 11 fluvoxamine, 2898 other SSRIs) compared with 80183 matched untreated control patients and assessed the relative risk (RR) for mortality is its study population.^32^

The last selected study was an observational study of 402 hospitalized COVID‐19 pneumonia patients which compared the outcomes (mortality, respiratory failure, IL‐6 level) of patients receiving antidepressants in the course of hospital admission (34 patients: 27 SSRI, 6 SNRI, 1 venlafaxine + duloxetine) versus patients who did not receive any antidepressants (368 patients).^33^ To sum up the data, in all these 8 studies we appraised 93,349 positive COVID‐19 patients which 4827 of them received either an SSRI or an SNRI antidepressant therapy including 4671 SSRI consumption (Fluvoxamine: 949, Fluoxetine: 610) and 66 SNRI therapy (Part C of Supporting Information Materials, Table S1–S2).

Quality assessment of studies was done based on the NIH tool and the quality of all selected studies was evaluated to be good.

**Figure 2:**
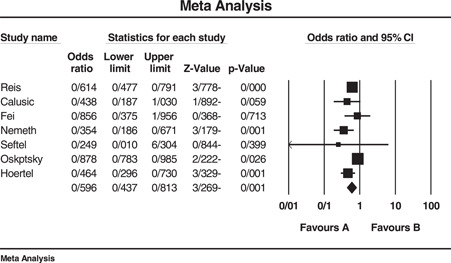
Forest plot of included studies investigating the effect of SSRI/SNRI on mortality of COVID‐19 patients. *I* ^2^ = 70.99, Eager's test (*p* value) = 0.052, sensitivity analysis = not significant. COVID‐19, coronavirus disease 2019; SNRI, serotonin‐norepinephrine reuptake inhibitor; SSRI, selective serotonin reuptake inhibitor

**Figure 3:**
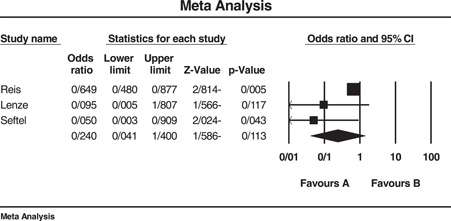
Forest plot of included studies investigating the effect of SSRI on hospitalization events of COVID‐19 patients. *I* ^2^ = 55.92, Eager's test (*p* value) = 0.096, Sensitivity analysis = not significant. COVID‐19, coronavirus disease 2019; SNRI, serotonin‐norepinephrine reuptake inhibitor; SSRI, selective serotonin reuptake inhibitor

Analysis of six studies for determining the overall effect of SSRI/SNRI on mortality of COVID‐19 patients revealed a significant OR equal to 0.596 (95% confidence interval [CI]: 0.437–0.813) with *I* ^2^ = 70.99. Three, two, and five studies were, respectively, used to determine the effect of fluvoxamine, fluoxetine, and fluvoxamine/fluoxetine on mortality of COVID‐19 patients that resulted in significant ORs of 0.595 (95% CI: 0.467–0.758), 0.620 (95% CI: 0.469–0.821), and 0.648 (95% CI: 0.550–0.765), respectively. Another analysis was performed on the effect of SSRIs on the hospitalization events of the patients that revealed a nonsignificant OR equal to 0.240 (95% CI: 0.041–1.4). The length of hospital stay was longer in patients who administrated SSRI although we could not statistically analyze this outcome. Egger's test for all analyses was nonsignificant. Due to the small number of studies available for assessment of fluoxetine's impact on mortality (two studies), performing Eggers' test was with no value and therefore was not done. The sensitivity of results to a specific study was not remarkable either. Figures 2 and 3 and part D of Supporting Information Materials illustrate the forest plot of the above‐mentioned analyses.

## 4 DISCUSSION

To the best of our knowledge, this is the first systematic review and meta‐analysis that explores the effect of SSRI/SNRI medications on COVID‐19 infection. Our analysis showed that SSRIs could significantly decrease mortality rate of COVID‐19 infected patients. Also, with nonsignificant statistical values, a decrease in hospitalization of patients was observed. However, in hospitalized patients, an increase in length of stay was noticed compared to controls.

Data extracted and analyzed after reviewing the final nine studies, reveal a reduction in mortality rate of COVID‐19 patients who consumed any of the SSRI or SNRI medications before COVID‐19 infection compared to controls. Comparing the impact of SSRIs and SNRIs use on mortality in treatment vs controls showed an overall OR of 0.596 (CI: 0.437–0.813, and *I* ^2^ = 70.99). The OR for risk of mortality in patients on fluvoxamine was 0.595 (CI: 0.467–0.758 and *I* ^2^ = 0.00), for fluoxetine was 0.620 (CI: 0.469–0.821, *I* ^2^ = 72.59) and for the two drugs being consumed together was estimated to be 0.648 (CI: 0.550–0.765, *I* ^2^ = 27.09). As mentioned before, only two studies reported the use of fluoxetine in COVID‐19 infection, and the higher OR reported for fluoxetine compared to fluvoxamine alone or in combination with fluvoxamine may be attributed to the small number of studies available for analysis. As an overall result, these two classes of antidepressants and specifically fluoxetine and fluvoxamine could decrease the likelihood of mortality in COVID‐19 patients, fluvoxamine showing superiority to fluoxetine. The analyses could not statistically demonstrate a significant decrease in hospitalization events (OR: 0.240, CI: 0.041–1.4, *I* ^2^ = 55.92) of the included patients but although statistically not significant, hospitalization was less than controls in the mentioned studies. Length of hospital stay was higher in the antidepressant‐treated patients in the studies evaluating this outcome.^29^, ^30^

Reis et al.^29^ reported part of the results of the TOGHETER trial, which was developed to assess different interventions such as consumption of hydroxychloroquine, metformin, and SSRIs on COVID‐19 outcomes in high‐risk outpatient subjects. After comparing 741 treated patients with 756 controls, the only significant clinical outcome was a lower rate of emergency setting visits in patients receiving fluvoxamine. All other findings including viral clearance, hospitalization, time to hospitalization, hospital length of stay, death, MV, and days on MV were not significantly different between the two groups.^29^ Another study discussed hospital stay and days on MV in ICU patients (51 cases/51 controls) treated with fluvoxamine. Although both of the outcomes were higher in numbers in the standard therapy group, the results were statistically not significant. The lower statistics in the treatment group could be attributed to the anti‐inflammatory effects of fluvoxamine. Although difference in hospital‐stay and length of MV was not significant, a significantly lower mortality rate (58.8% vs. 76.5%) with a hazard ratio of 0.58 was observed in the fluvoxamine arm.^29^, ^30^ Lenze et al.^13^ investigated an index called clinical deterioration in out‐patient COVID‐19 cases in an RCT and divided the patients into the treatment arm (receiving fluvoxamine) and the control arm. This index was defined as having shortness of breath, O_2_ saturation less than 92%, or hospitalization due to these two conditions. Six patients fulfilled this index in the placebo group while none of the patients in the treatment arm had any of the criteria mentioned.^13^ An interesting study, investigates the OR of being infected by COVID‐19 in patients of a psychiatric care hospital comparing patients on antidepressants with those not receiving antidepressants. In patients generally receiving antidepressants, not specifically defining the drug class, a 72% less probability of COVID‐19 infection was observed and 70% lower rate of infection was reported for patients specifically on SSRI/SNRI treatment.^34^ Hoertel et al. performed a large‐scale study on hospitalized COVID‐19 patients and investigated the effect of exposure to antidepressants and the risk of intubation or death as a primary endpoint. This retrospective study illustrated lower intubation and/or death among patients who were exposed to anti‐depressants, including SSRIs and SNRIs both in patients with ward and ICU admissions. Interestingly, psychiatric patients with at least a 3‐month history of antidepressant use before admission following COVID‐19 infection had a higher risk of intubation/death. Also, the combination of antidepressants had no effect on the risk of intubation or death.^15^ Confirming previous studies, Fei et al.^33^ also found lower mortality rates in patients who were treated with SSRI and/or SNRI. This study pointed out that acute respiratory distress syndrome and endotracheal intubation were significantly lower in antidepressant‐treated patients although respiratory failure was not significantly different. The largest study with the largest sample size was a retrospective cohort that included 83,548 patients of which 3401 were exposed to SSRIs. A propensity score matching was performed between treated and control patients to diminish confounding factors. The study reported an 8% reduction in RR of mortality in patients who used any kind of SSRI medication, a 28% reduction in patients for whom fluoxetine was prescribed, and a 26% reduction in the group of patients using either fluoxetine or fluvoxamine.^32^ The last study was a prospective cohort that congruent with other studies showed significantly lower mortality rate, ICU admission, and MV in fluvoxamine‐treated patients. A remarkable finding of this study was that on day 14 of the infection, no COVID‐19‐related symptoms were detectable in the treatment group taking into account that 60% of non‐fluvoxamine‐treated patients presented with symptoms of infection^31^ A very recent study reported findings that showed no benefit in using SSRIs contrary to the results of Hoertel et al.^15^ In this retrospective study, 832 patients on SSRIs and 8212 patients without prior use of SSRIs were compared and no significant odds of reducing mortality was found.^35^ This finding although at first glance may appear contrary to the results of this meta‐analysis, but it is in line with our overall results and the results of Hoertel et al.,^15^ showing that long‐term use of antidepressants probably in patients with a pre‐existing psychiatric disease may not be as beneficial for COVID‐19 infected patients or in other words, their baseline clinical condition may outweigh the beneficial effects of antidepressant use. As suggested previously by Hoertel et al.^15^ these patients may be having biological markers, T‐cell mediated immunity dysfunction or comorbidities that may deteriorate their infection process and, therefore, lead to a higher rate of mortality. Therefore, the results of this study can only propose that the use of SSRI/SNRI in the period of COVID‐19 infection may benefit patients in terms of reducing the mortality rate of COVID‐19 infection.

Although fluvoxamine has been found superior in preventing complications of COVID‐19 infection, it is noteworthy that caution must be taken in the use of this medication in patients with cardiovascular disease and unstable seizure. However, short‐term use for reducing the risk of COVID‐19 infection severity, may most likely be safe.^36^, ^37^ A scoping review on the exposure of patients to psychotropic drugs reached interesting and congruent results. Bonnet et al.^38^ reached a conclusion that exposure to SSRI or SNRI drugs may reduce mortality rate of patients. This study found that other psychiatric drugs such as trazodone may have an effect on reducing infection rate. Another review on the effect of SSRIs on COVID‐19 concluded that these drugs may have a substantial effect on reducing mortality and hospitalization; although our results were not statistically significant considering the effect of SSRI/SNRI on hospitalization of COVID‐19 patients.^39^

Three studies have investigated risk factors for mortality, intubation/death and moderation of treatment. Reis et al.^29^ found no moderator of treatment among age, gender, body mass index, onset of symptoms, cardiovascular disease, corticosteroid use, chronic kidney disease, and smoking. Contrary to the previous study, Hoertel et al.^17^ reported a significant hazard ratio for the association of some baseline characteristics of the patients and death/intubation. Obesity, male gender, older age, smoking, and underlying disease increase risk of severe COVID‐19 and history of psychiatric disease were presented among the risk factors. Another study led to a conclusion that fluvoxamine's impact on mortality was more significant in women rather than men relevant to the fact that published articles on mortality of COVID‐19 have also reported higher mortality rates among men.^30^, ^40^ This difference has been attributed to probable hormonal differences. Lower testosterone levels have been linked to increased inflammatory processes and estrogen has shown protective quality by showing anti‐inflammatory effects.^40^, ^41^ This article described age, coronary artery disease, continuous renal replacement therapy, and fluvoxamine consumption as significant variables in determining the prognosis of COVID‐19 infected patients.^30^

### 4.1 Possible mechanisms suggested for protective effects of SSRI/SNRIs on COVID‐19 infection

Although the pure mechanism of the above‐mentioned effects has not been fully discovered, several explanations have been reported in the literature. A meta‐analysis and systematic review was conducted before the outbreak of COVID‐19 that proposed the anti‐inflammatory effects of SSRIs. This study reported a significant decrease in the level of IL‐6, tumor necrosis factor‐alpha, IL‐10, and C‐C motif ligand 2 chemokine as inflammatory markers.^19^ Another theory is the antisphingomyelinase effect of antidepressants. Sphingomyelinase leads to ceramide production that is accounted as a receptor for viruses. The antisphingomyelinase property of antidepressants can therefore functionally inhibit entry of coronavirus into the human cells.^42^, ^43^ An in vitro experiment was performed on the effect of fluoxetine on the inhibition of coronavirus. This study reported a successful inhibitory effect of fluoxetine on the gene expression of this virus.^44^ Fluvoxamine may also have an immunomodulatory effect by showing high affinity for sigma 1 receptor (S1R).^45^ S1R has shown significant roles in modulating the immune system and inhibition of inflammatory cytokines in vitro.^46^, ^47^, ^48^ Fluoxetine and fluvoxamine have shown to affect intravesicular pH thereby impairing the fusion of virus compartments and its internal spread.^49^ Reduced platelet aggregation and degranulation of mast cells are other proposed mechanisms for fluvoxamine and its role in the reducing inflammation and mortality and improvement of patient outcomes in COVID‐19 infection.^16^, ^50^

### 4.2 The rationale for using SSRI/SNRI drugs for prevention of severe COVID‐19 infection

Although SSRI/SNRI drugs were first introduced as mood stabilizers, their indication now has been extended to the treatment of other disorders.^51^, ^52^, ^53^, ^54^ Considering the proposed mechanisms, it is somehow logical to consider these medications as an adjunct in the treatment of infectious diseases such as COVID‐19. According to a report, a 1‐month cost of an SSRI/SNRI will be an average of 75–320$ depending on the medication, considering it a manageable cost for a single duration of therapy.^55^, ^56^ Comparingly speaking a 5‐day treatment course with remdesivir costs 2340$ which was recently introduced to prevent high‐risk patients from developing severe infection.^56^, ^57^ Although SSRI/SNRIs have a range of adverse effects like all other medications, their adverse effects are variable between different populations and are mostly transient and leave no sequala for the patients in particular when used for a short duration of time.^58^ Overall, SSRI/SNRI are safe, inexpensive, and effective drugs that may benefit COVID‐19 patients before it is too late. Although these drugs are available in most parts of the world as psychiatric medications, providing extra dosage for treatment of COVID‐19 needs multi‐level and national‐wide governance and cooperation with pharmaceutical companies to avoid shortages.^59^ It is worth mentioning that although these drugs may reduce mortality of patients, they could not substitute vaccination as a preventive strategy. Regardless of the effect of vaccination on preventing COVID‐19, all proposed preventive strategies are aimed at reducing the socioeconomic burden of this disease.^60^

### 4.3 Limitation

The major limitation of this study was the scarcity of available data on antidepressant use in COVID‐19 infection, specifically RCTs. The source of heterogeneity for this meta‐analysis may be patients with variable severity of infection, the different setting and design of the included studies, and differences in the type of antidepressants used with variable doses in different studies. Variability in dosing of the antidepressant used was seen in different studies and, therefore, further studies are required to establish the correct and efficient dosing of the specific antidepressant used. To minimize the destructive effects of heterogeneity in the available studies, a subgroup analysis was performed.

## 5 CONCLUSION

Our study suggests a beneficial effect of SSRI/SNRIs on decreasing mortality rate in COVID‐19 infected patients, suggesting the superiority of fluvoxamine to fluoxetine in such patients. Considering their safe profile and comparingly low cost for a short duration of treatment, these medications may be helpful not only to prevent clinical complications of COVID‐19 infection but also further financial burden on the health care systems and also help towards alleviating complexity of care in this ongoing pandemic. Aside from their effectiveness, SSRI/SNRI administration has some limitations such as their onset of action and reaching therapeutic levels regarding their half‐life. Also concerns remain for their exact dosing, interval of dosing and time of starting administration in this specific clinical condition, that all require detailed kinetic and clinical studies in large study populations. Additionally, the cost‐effectiveness of the prescription of such drugs according to the aforementioned issues should be investigated in more depth.^61^

## AUTHOR CONTRIBUTIONS

*Conceptualization*: Dena Firouzabadi, Fatemeh Kheshti, Saeed Abdollahifard, Erfan Taherifard, Mohammad Reza Kheshti. *Formal analysis*: Fatemeh Kheshti, Saeed Abdollahifard. *Investigation*: Fatemeh Kheshti, Saeed Abdollahifard, Erfan Taherifard, Mohammad Reza Kheshti. *Methodology*: Dena Firouzabadi, Fatemeh Kheshti, Saeed Abdollahifard, Erfan Taherifard, Mohammad Reza Kheshti. *Supervision*: Dena Firouzabadi. *Writing – original draft preparation*: Dena Firouzabadi, Fatemeh Kheshti, Saeed Abdollahifard, Erfan Taherifard, Mohammad Reza Kheshti. *Writing – review & editing*: Dena Firouzabadi, Fatemeh Kheshti, Saeed Abdollahifard, Erfan Taherifard, Mohammad Reza Kheshti. All authors have read and approved the final version of the manuscript.

## CONFLICTS OF INTEREST

The authors declare no conflicts of interest.

## TRANSPARENCY STATEMENT

The lead author Fatemeh Kheshti affirms that this manuscript is an honest, accurate, and transparent account of the study being reported; that no important aspects of the study have been omitted; and that any discrepancies from the study as planned (and, if relevant, registered) have been explained.

## Supporting information

Supporting information.

## Data Availability

The data that supports the findings of this study are available in the Supporting Information Materials of this article.

## References

[1] Guo Y‐R , Cao Q‐D , Hong Z‐S , et al. The origin, transmission and clinical therapies on coronavirus disease 2019 (COVID‐19) outbreak—an update on the status. Mil Med Res. 2020;7(1):11. 32169119 10.1186/s40779-020-00240-0 PMC7068984

[2] Kaeuffer C , Le Hyaric C , Fabacher T , et al. Clinical characteristics and risk factors associated with severe COVID‐19: prospective analysis of 1,045 hospitalised cases in North‐Eastern France, March 2020. Euro Surveill. 2020;25(48):2000895. 33272355 10.2807/1560-7917.ES.2020.25.48.2000895 PMC7716399

[3] Coccia M . Meta‐analysis to explain unknown causes of the origins of SARS‐COV‐2. Environ Res. 2022;211:113062. 35259407 10.1016/j.envres.2022.113062 PMC8897286

[4] Lotfi M , Hamblin MR , Rezaei N . COVID‐19: transmission, prevention, and potential therapeutic opportunities. Clin Chim Acta. 2020;508:254‐66. 32474009 10.1016/j.cca.2020.05.044 PMC7256510

[5] Stufano A , Lisco S , Bartolomeo N , et al. COVID‐19 outbreak in Lombardy, Italy: An analysis on the short‐term relationship between air pollution, climatic factors and the susceptibility to SARS‐CoV‐2 infection. Environ Res. 2021;198:111197. 10.1016/j.envres.2021.111197 PMC8078046 33930404

[6] Núñez‐Delgado A , Bontempi E , Coccia M , Kumar M , Farkas K , Domingo JL . SARS‐CoV‐2 and other pathogenic microorganisms in the environment. Environ Res. 2021;201:111606. 34181924 10.1016/j.envres.2021.111606 PMC8459334

[7] Coccia M . Factors determining the diffusion of COVID‐19 and suggested strategy to prevent future accelerated viral infectivity similar to COVID. Sci Total Environ. 2020;729:138474. 32498152 10.1016/j.scitotenv.2020.138474 PMC7169901

[8] Beigel JH , Tomashek KM , Dodd LE , et al. Remdesivir for the treatment of Covid‐19—final report. N Engl J Med. 2020;383(19):1813‐1826. 32445440 10.1056/NEJMoa2007764 PMC7262788

[9] Gautret P , Lagier JC , Parola P , et al. Hydroxychloroquine and azithromycin as a treatment of COVID‐19: results of an open‐label non‐randomized clinical trial. Int J Antimicrob Agents. 2020;56(1):105949. 32205204 10.1016/j.ijantimicag.2020.105949 PMC7102549

[10] Skipper CP , Pastick KA , Engen NW , et al. Hydroxychloroquine in nonhospitalized adults with early COVID‐19: a randomized trial. Ann Intern Med. 2020;173(8):623‐631. 32673060 10.7326/M20-4207 PMC7384270

[11] Doi Y , Hibino M , Hase R , et al. A prospective, randomized, open‐label trial of early versus late favipiravir therapy in hospitalized patients with COVID‐19. Antimicrob Agents Chemother. 2020;64(12):e01897‐20. 32958718 10.1128/AAC.01897-20 PMC7674035

[12] Tomazini BM , Maia IS , Cavalcanti AB , et al. Effect of dexamethasone on days alive and ventilator‐free in patients with moderate or severe acute respiratory distress syndrome and COVID‐19: the CoDEX randomized clinical trial. JAMA. 2020;324(13):1307‐1316. 32876695 10.1001/jama.2020.17021 PMC7489411

[13] Lenze EJ , Mattar C , Zorumski CF , et al. Fluvoxamine vs placebo and clinical deterioration in outpatients with symptomatic COVID‐19: a randomized clinical trial. JAMA. 2020;324(22):2292‐2300. 33180097 10.1001/jama.2020.22760 PMC7662481

[14] Németh ZK , Szûcs A , Vitrai J , Juhász D , Németh JP , Holló A . Fluoxetine use is associated with improved survival of patients with COVID‐19 pneumonia: a retrospective case‐control study. Ideggyogy Sz. 2021;74(11‐12):389‐396. 34856085 10.18071/isz.74.0389

[15] Hoertel N , Sánchez‐Rico M , Vernet R , et al. Association between antidepressant use and reduced risk of intubation or death in hospitalized patients with COVID‐19: results from an observational study. Mol Psychiatry. 2021;26(9):5199‐5212. 33536545 10.1038/s41380-021-01021-4

[16] Sukhatme VP , Reiersen AM , Vayttaden SJ , Sukhatme VV . Fluvoxamine: a review of its mechanism of action and its role in COVID‐19. Front Pharmacol. 2021;12:652688. 33959018 10.3389/fphar.2021.652688 PMC8094534

[17] Hoertel N . Do the selective serotonin reuptake inhibitor antidepressants fluoxetine and fluvoxamine reduce mortality among patients with COVID‐19? JAMA Network Open. 2021;4(11):e2136510. 34779851 10.1001/jamanetworkopen.2021.36510

[18] Kornhuber J , Hoertel N , Gulbins E . The acid sphingomyelinase/ceramide system in COVID‐19. Mol Psychiatry. 2021;27:1‐8. 34608263 10.1038/s41380-021-01309-5 PMC8488928

[19] Köhler CA , Freitas TH , Stubbs B , et al. Peripheral alterations in cytokine and chemokine levels after antidepressant drug treatment for major depressive disorder: systematic review and meta‐analysis. Mol Neurobiol. 2018;55(5):4195‐4206 28612257 10.1007/s12035-017-0632-1

[20] Słuzewska A , Rybakowski JK , Laciak M , Mackiewicz A , Sobieska M , Wiktorowicz K . Interleukin‐6 serum levels in depressed patients before and after treatment with fluoxetine. Ann N Y Acad Sci. 1995;762:474‐476. 7668562 10.1111/j.1749-6632.1995.tb32372.x

[21] Schloer S , Brunotte L , Goretzko J , et al. Targeting the endolysosomal host‐SARS‐CoV‐2 interface by clinically licensed functional inhibitors of acid sphingomyelinase (FIASMA) including the antidepressant fluoxetine. Emerg Microbes Infect. 2020;9(1):2245‐2255. 32975484 10.1080/22221751.2020.1829082 PMC7594754

[22] Creeden JF , Imami AS , Eby HM , et al. Fluoxetine as an anti‐inflammatory therapy in SARS‐CoV‐2 infection. Biomed Pharmacother. 2021;138:111437. 33691249 10.1016/j.biopha.2021.111437 PMC7904450

[23] McCloskey DJ , Postolache TT , Vittone BJ , et al. Selective serotonin reuptake inhibitors: measurement of effect on platelet function. Transl Res. 2008;151(3):168‐172. 18279816 10.1016/j.trsl.2007.10.004 PMC2391274

[24] Chen ZH , Xiao L , Chen JH , et al. Effects of fluoxetine on mast cell morphology and protease‐1 expression in gastric antrum in a rat model of depression. World J Gastroenterol. 2008;14(45):6993‐6998. 19058337 10.3748/wjg.14.6993 PMC2773865

[25] Xu S , Huang R , Sy LSEA . COVID‐19 vaccination and non–COVID‐19 mortality risk—seven integrated health care organizations, United States, December 14, 2020–July 31, 2021. MMWR Morb Mortal Wkly Re. 10.15585/mmwr.mm7043e2 PMC8553028 34710075

[26] Facente SN , Reiersen AM , Lenze EJ , Boulware DR , Klausner JD . Fluvoxamine for the early treatment of SARS‐CoV‐2 infection: a review of current evidence. Drugs. 2021;81(18):2081‐2089. 34851510 10.1007/s40265-021-01636-5 PMC8633915

[27] Page MJ , McKenzie JE , Bossuyt PM , et al. The PRISMA 2020 statement: an updated guideline for reporting systematic reviews. BMJ. 2021;372:n71. 33782057 10.1136/bmj.n71 PMC8005924

[28] Health NIo. Study quality assessment tools . 2021. URL: https://www-nhlbi-nih-gov/health-topics/study-quality-assessment-tools.2021

[29] Reis G , dos Santos Moreira‐Silva EA , Silva DCM , et al. Effect of early treatment with fluvoxamine on risk of emergency care and hospitalisation among patients with COVID‐19: the TOGETHER randomised, platform clinical trial. The Lancet Global Health. 2022;10:42. 10.1016/S2214-109X(21)00448-4 PMC8550952 34717820

[30] Calusic M , Marcec R , Luksa L , et al. Safety and efficacy of fluvoxamine in COVID‐19 ICU patients: an open label, prospective cohort trial with matched controls. Br J Clin Pharmacol. 2021;88:2065‐2073. 34719789 10.1111/bcp.15126 PMC8653355

[31] Seftel D , Boulware DR . Prospective cohort of fluvoxamine for early treatment of coronavirus disease 19. Open Forum Infect Dis. 2021;8(2):ofab050. 33623808 10.1093/ofid/ofab050 PMC7888564

[32] Oskotsky T , Maric I , Tang A , et al. Mortality risk among patients with COVID‐19 prescribed selective serotonin reuptake inhibitor antidepressants. JAMA Netw Open. 2021;4(11):e2133090. 34779847 10.1001/jamanetworkopen.2021.33090 PMC8593759

[33] Fei L , Santarelli G , D'anna G , et al. Can SSRI/SNRI antidepressants decrease the ‘cytokine storm’ in the course of COVID‐19 pneumonia? Panminerva Med. 2021. 10.23736/S0031-0808.21.04436-0 34240839

[34] Clelland CL , Ramiah K , Steinberg L , Clelland JD . Analysis of the impact of antidepressants and other medications on COVID‐19 infection risk in a chronic psychiatric in‐patient cohort. BJPsych Open. 2021;8(1):e6. 34859759 10.1192/bjo.2021.1053 PMC8649363

[35] Rauchman SH , Mendelson SG , Rauchman C , Kasselman LJ , Pinkhasov A , Reiss AB . Ongoing use of SSRIs does not alter outcome in hospitalized COVID‐19 patients: a retrospective analysis. J Clin Med. 2021;11(1):70. 35011811 10.3390/jcm11010070 PMC8745642

[36] Kim KY , Craig JM , Hawley JM . Seizure possibly associated with fluvoxamine. Ann Pharmacother. 2000;34(11):1276‐1278. 11098342 10.1345/aph.10134

[37] Roos JC . Cardiac effects of antidepressant drugs. A comparison of the tricyclic antidepressants and fluvoxamine. Br J Clin Pharmacol. 1983;15(Suppl 3):439s‐445s. 6407505 10.1111/j.1365-2125.1983.tb02135.x PMC1427661

[38] Bonnet U , Juckel G . COVID‐19 outcomes: does the use of psychotropic drugs make a difference? accumulating evidence of a beneficial effect of Antidepressants‐A scoping review. J Clin Psychopharmacol. 2022;42(3):284‐292. 35420565 10.1097/JCP.0000000000001543 PMC9042214

[39] Foletto VS , da Rosa TF , Serafin MB , Hörner R . Selective serotonin reuptake inhibitor (SSRI) antidepressants reduce COVID‐19 infection: prospects for use. Eur J Clin Pharmacol. 2022;78:1‐11. 35943535 10.1007/s00228-022-03372-5 PMC9360648

[40] Moiseev S , Brovko M , Tao E , Bulanov N , Akulkina L , Fomin V . Sex differences in mortality in the intensive care unit patients with severe COVID‐19. J Infect. 2021;82(2):282‐327. 10.1016/j.jinf.2020.09.031 PMC7521429 32998037

[41] Al‐Lami RA , Urban RJ , Volpi E , Algburi AMA , Baillargeon J . Sex hormones and novel corona virus infectious disease (COVID‐19). Mayo Clin Proc. 2020;95(8):1710‐1714. 32753145 10.1016/j.mayocp.2020.05.013 PMC7256539

[42] Carpinteiro A , Edwards MJ , Hoffmann M , et al. Pharmacological inhibition of acid sphingomyelinase prevents uptake of SARS‐CoV‐2 by epithelial cells. Cell Rep Med. 2020;1(8):100142. 33163980 10.1016/j.xcrm.2020.100142 PMC7598530

[43] Gulbins A , Schumacher F , Becker KA , et al. Antidepressants act by inducing autophagy controlled by sphingomyelin‐ceramide. Mol Psychiatry. 2018;23(12):2324‐2346. 30038230 10.1038/s41380-018-0090-9 PMC6294742

[44] Zimniak M , Kirschner L , Hilpert H , Seibel J , Bodem J . The serotonin reuptake inhibitor Fluoxetine inhibits SARS‐CoV‐2. bioRxiv. 2020:2020.06.14.150490. 10.1038/s41598-021-85049-0 PMC7961020 33723270

[45] Mouffak S , Shubbar Q , Saleh E , El‐Awady R . Recent advances in management of COVID‐19: a review. Biomed Pharmacother. 2021;143:112107. 34488083 10.1016/j.biopha.2021.112107 PMC8390390

[46] Rosen DA , Seki SM , Fernández‐Castañeda A , et al. Modulation of the sigma‐1 receptor‐IRE1 pathway is beneficial in preclinical models of inflammation and sepsis. Sci Transl Med. 2019;11(478):eaau5266. 30728287 10.1126/scitranslmed.aau5266 PMC6936250

[47] Szabo A , Kovacs A , Frecska E , Rajnavolgyi E . Psychedelic N,N‐dimethyltryptamine and 5‐methoxy‐N,N‐dimethyltryptamine modulate innate and adaptive inflammatory responses through the sigma‐1 receptor of human monocyte‐derived dendritic cells. PLoS One. 2014;9(8):e106533. 25171370 10.1371/journal.pone.0106533 PMC4149582

[48] Hashimoto Y , Suzuki T , Hashimoto K . Old drug fluvoxamine, new hope for COVID‐19. Eur Arch Psychiatry Clin Neurosci. 2021;272:1‐3. 10.1007/s00406-021-01326-z PMC8412866 34476589

[49] Homolak J , Kodvanj I . Widely available lysosome targeting agents should be considered as potential therapy for COVID‐19. Int J Antimicrob Agents. 2020;56(2):106044. 32522674 10.1016/j.ijantimicag.2020.106044 PMC7275137

[50] Anderson GM . Fluvoxamine, melatonin and COVID‐19. Psychopharmacology (Berl). 2021;238(2):611. 33392622 10.1007/s00213-020-05753-z PMC7779245

[51] Ghaffari Darab M , Hedayati A , Khorasani E , Bayati M , Keshavarz K . Selective serotonin reuptake inhibitors in major depression disorder treatment: an umbrella review on systematic reviews. Int J Psychiatry Clin Pract. 2020;24(4):357‐370. 32667275 10.1080/13651501.2020.1782433

[52] Sathianathen NJ , Hwang EC , Mian R , et al. Selective serotonin re‐uptake inhibitors for premature ejaculation in adult men. Cochrane Database Syst Rev. 2021;3(3):Cd012799. 33745183 10.1002/14651858.CD012799.pub2 PMC8094926

[53] Wang F , Wang J , Cao Y , Xu Z . Serotonin‐norepinephrine reuptake inhibitors for the prevention of migraine and vestibular migraine: a systematic review and meta‐analysis. Reg Anesth Pain Med. 2020;45(5):323‐330. 32205412 10.1136/rapm-2019-101207

[54] Aiyer R , Barkin RL , Bhatia A . Treatment of neuropathic pain with venlafaxine: a systematic review. Pain Med. 2017;18(10):1999‐2012. 27837032 10.1093/pm/pnw261

[55] Rockville (MD): Agency for Healthcare Research and Quality (US) ; 2007‐. [Table], Dose & Price of Selected Antidepressants. Available from: https://www.ncbi.nlm.nih.gov/books/NBK43419/table/clindep.t1/ECaOHSUCAfACsGAICERSGfCI

[56] Carta A , Conversano C . Cost utility analysis of Remdesivir and Dexamethasone treatment for hospitalised COVID‐19 patients–a hypothetical study. BMC Health Serv Res. 2021;21(1):986. 34537034 10.1186/s12913-021-06998-w PMC8449700

[57] Gottlieb RL , Vaca CE , Paredes R , et al. Early Remdesivir to prevent progression to severe Covid‐19 in outpatients. N Engl J Med. 2021;386(4):305‐315. 34937145 10.1056/NEJMoa2116846 PMC8757570

[58] Ferguson JM . SSRI antidepressant medications: adverse effects and tolerability. Prim Care Companion J Clin Psychiatry. 2001;3(1):22‐27. 15014625 10.4088/pcc.v03n0105 PMC181155

[59] Coccia M . Pandemic prevention: lessons from COVID‐19. Encyclopedia. 2021;1(2):433‐444.

[60] Benati I , Coccia M . Global analysis of timely COVID‐19 vaccinations: improving governance to reinforce response policies for pandemic crises. International Journal of Health Governance. 2022;27(3):240‐253.

[61] Le Corre P , Loas G . Difficulty in repurposing selective serotonin reuptake inhibitors and other antidepressants with functional inhibition of acid sphingomyelinase in COVID‐19 infection. Front Pharmacol. 2022;13:849095. 35308205 10.3389/fphar.2022.849095 PMC8927035

