# Supplementary material for: The effect of selective serotonin and norepinephrine reuptake inhibitors on clinical outcome of COVID‐19 patients: A systematic review and meta‐analysis
Source: Health Sci Rep. 2022 Oct 17;5(6):e892. doi: 10.1002/hsr2.892 (PMC9577115; doi:10.1002/hsr2.892)
Supplement: Supplementary file 1 — Supporting information. [file HSR2-5-0-s001.docx]

**Supplementary materials**

**Part A: Search strategy**

**Part B: Number of retrieved articles from each database**

**Part C: Tables of characteristics and outcomes of included studies**

**Part D: Forest plots**

**Part A: Search strategy**

(((((((((((((((((((((COVID 19[MeSH Terms]) OR (Covid-19[Title/Abstract])) OR (SARS-CoV-2 Infection[Title/Abstract])) OR (SARS CoV 2 Infection[Title/Abstract])) OR (2019 Novel Coronavirus Disease[Title/Abstract])) OR (2019 Novel Coronavirus Infection[Title/Abstract])) OR (2019-nCoV Disease[Title/Abstract])) OR (2019 nCoV Disease[Title/Abstract])) OR (COVID-19 Virus Infection[Title/Abstract])) OR (Coronavirus Disease 2019[Title/Abstract])) OR (Coronavirus Disease-19[Title/Abstract])) OR (Coronavirus Disease 19[Title/Abstract])) OR (Severe Acute Respiratory Syndrome Coronavirus 2 Infection[Title/Abstract])) OR (SARS Coronavirus 2 Infection[Title/Abstract])) OR (COVID-19 Virus Disease[Title/Abstract])) OR (COVID 19 Virus Disease[Title/Abstract])) OR (2019-nCoV Infection[Title/Abstract])) OR (2019 nCoV Infection[Title/Abstract])) OR (COVID19[Title/Abstract])) OR (COVID-19 Pandemic[Title/Abstract])) OR (COVID 19 Pandemic[Title/Abstract])) AND (((((((((((((((((((((Serotonin Uptake Inhibitors[MeSH Major Topic]) OR (5-Hydroxytryptamine Uptake Inhibitor[Title/Abstract])) OR (Serotonin Reuptake Inhibitor[Title/Abstract])) OR (Serotonin Uptake Inhibitor[Title/Abstract])) OR (5-HT Uptake Inhibitor[Title/Abstract])) OR (Selective Serotonin Reuptake Inhibitor[Title/Abstract])) OR (Citalopram[Title/Abstract])) OR (Escitalopram[Title/Abstract])) OR (Fluoxetine[Title/Abstract])) OR (Paroxetine[Title/Abstract])) OR (Sertraline[Title/Abstract])) OR (SSRI[Title/Abstract])) OR (Serotonin and Noradrenaline Reuptake Inhibitors[MeSH Major Topic])) OR (Serotonin[Title/Abstract] AND Norepinephrine Reuptake Inhibitors[Title/Abstract])) OR (Serotonin[Title/Abstract] AND Noradrenaline Uptake Inhibitors[Title/Abstract])) OR (Serotonin[Title/Abstract] AND Norepinephrine Uptake Inhibitors[Title/Abstract])) OR (SNRI[Title/Abstract])) OR (desvenlafaxine[Title/Abstract])) OR (Duloxetine[Title/Abstract])) OR (levomilnacipran[Title/Abstract])) OR (venlafaxine[Title/Abstract]))

**Part B: Number of retrieved articles from each database**

**PubMed: 91 Articles**

**Scopus: 91 Articles**

**Embase: 111 Articles**

**Part C: Tables of characteristics and outcomes of included studies.**

Table 1. Characteristics of included studies investigating the effect of SSRI/SNRI medications on patients with COVID-19

| Author | Design | Number of patients  *Case/control (based on their design) | Population | Intervention/ placebo  Or exposure | Outcomes |
| --- | --- | --- | --- | --- | --- |
| Reis et al | Double blind randomized clinical trial | 741/756 | High risk Brazilian adults infected by COVID-19 | Fluvoxamine 100mg twice a day for 10 days / Standard treatment | -Hospital admission event  -Mortality  -length of hospital stay  -Days on MV  -MV  Viral clearance  -ER visit |
| Calusic et al | Open-label, prospective cohort trial | 51/51 | COVID-19 ICU patients | Fluvoxamine 100mg three times a day for 15 days / Standard treatment | -length of hospital and ICU stay  -Mortality  -Days on MV |
| Lenze et al | Double blind randomized clinical trial | 80/72 | COVID-19 non-hospitalized patients | Fluvoxamine 100mg three times a day for 15 days / Standard treatment | -Clinical deterioration  (Shortness of breath or hospitalization due to it O2 saturation less than 92% or these symptoms lead to hospitalization)  -Mortality  -Hospitalization |
| Clelland et al | Retrospective cohort | Total 165  Antidepressant:38 patients | Psychiatric hospital patients | Antidepressant drugs including SSRI, SNRI and SARI in combination with other psychiatric drugs. | Being infected by COVID-19 |
| Hoertel et al | Retrospective cohort | Total : 7230  SSRI:195  Citalopram:21  Escitalopram:63  Fluoxetine:30  Fluvoxamine:1  Paroxetine:63  Serteraline:22  Vortioxetine:2  SNRI:59 | Hospitalized COVID-19 patients | Antidepressant drugs | Death and intubation |
| Fei et al | Observational study | 34/368  Antidepressant:34  Serteraline:9  Escitalopram:8  Citalopram:5  Paroxetine:5  Venlafaxine:3  Duloxetine:3  SSRI+SNRI:1 | Hospitalized COVID-19 patients | SNRI and/or SSRI | -Death  -Respiratory failure and ARDS  -IL-6 level  -Intubation  -ICU admission  -CCI score  -Non invasive ventilation |
| Nemeth et al | Retrospective case-control | 269  Received fluoxetine:110  No fluoxetine recived:159 | Hospitalized moderate to severe COVID-19 patients with COVID-19 pneumonia | Fluoxetine 20mg per day | Mortality |
| Oskotsky et al | Retrospective cohort | 83584  SSRI:3401  Fluoxetine:470  Fluoxetine or fluvoxamine:481  Other SSRI:2898 | COVID-19 patients | SSRI | Mortality |
| Seftel et al | Prospective cohort | 114  Fluvoxamine treated: 65  Others: 48 | COVID-19 patients | Fluvoxamine 50mg twice a day for 14 days | Mortality  Hospital admission |

SSRI: Selective Serotonin Reuptake Inhibitor

SNRI: Selective Norepinephrine Reuptake Inhibitor

SARI: Serotonin Antagonist and Reuptake Inhibitors
MV: Mechanical Ventilation

ER: Emergency Room

ICU: Intensive Care Unit

ARDS: Acute Respiratory Distress Syndrome

IL6: Interleukin 6

*Median(IQR)

Table 2. Outcomes of included studies investigating the effect of SSRI/SNRI on patients with COVID-19

| Author | Subgroups | Hospitalization  (number of patients (percentage)) | Mortality  (number of patients (percentage)) | Length of hospital stay  (mean days) | Being affected by COVID-19  (Odds ratio with 95% CI) |
| --- | --- | --- | --- | --- | --- |
| Reis et al | Cases | 79(11%) | 17(2%) | 8(5-13)* |  |
|  | Controls | 119(16%) | 25(3%) | 6 (3-10.75)* |  |
| Calusic et al | Cases |  | 30(58.8%) | 20.98 |  |
|  | Controls |  | 39(76.5%) | 17.04 |  |
| Lenze et al | Cases | 0 | 0 |  |  |
|  | Controls | 4(5.55%) | 0 |  |  |
| Clelland et al | Antidepressant  (unadjusted) |  |  |  | 0.327(0.153-0.698) |
|  | Antidepressant  (Fully adjusted) |  |  |  | 0.280(0.094-0.837) |
|  | SSRI/SNRI  (unadjusted) |  |  |  | 0.302(0.120-0.780) |
| Hoertel et al | Any antidepressant  (matched) |  | 22% |  |  |
|  | Controls for any antidepressant  (matched) |  | 34.2% |  |  |
|  | SSRI(Matched) |  | 21% |  |  |
|  | Controls for SSRI |  | 36.4% |  |  |
|  | SNRI(Matched) |  | 22% |  |  |
|  | Controls for SNRI(Matched) |  | 42.2% |  |  |
| Fei et al | AD-treated |  | 8(23.5%) |  |  |
|  | Others |  | 97(26.4% |  |  |
| Nemeth et al | Treated with fluoxetine |  | 15(13.6%) |  |  |
|  | Standard treatment |  | 49(30.8%) |  |  |
| Oskotsky | Exposed to any SSRI |  | 497(14.6%)  *0.92(0.85-0.99) |  |  |
|  | Matched control for exposed to any SSRI |  | 1107(16.3%) |  |  |
|  | Exposed to fluoxetine |  | 46(9.8%)  *0.72(0.54-0.97) |  |  |
|  | Matched control for exposed to fluoxetine |  | 937(13.3% |  |  |
|  | Exposed to fluoxetine or Fluvoxamine |  | 48(10.0%)  *0.74(0.55-0.99) |  |  |
|  | Matched control for exposed to fluoxetine or Fluvoxamine |  | 956(13.3%) |  |  |
|  | Exposed to other SSRI(not fluoxetine or Fluvoxamine) |  | 447(15.4)  *0.92(0.84-1.00) |  |  |
|  | Matched control for exposed to other SSRI(not fluoxetine or Fluvoxamine) |  | 1474(17.0) |  |  |
| Seftel et al | Fluvoxamine treated | 0(0) | 0 |  |  |
|  | Others | 6(12.5%) | 1(2%) |  |  |

#HR and 95% CI

*RR and 95% CI

SSRI: Selective Serotonin Reuptake Inhibitor

SNRI: Selective Norepinephrine Reuptake Inhibitor

AD: Antidepressant

**Part D: Forest plots**

Figure 1. Forest plot of included studies investigating the effect of Fluvoxamin on mortality of COVID-19 patients. I^2^ =0.00, Eager’s test (P value) =0.1966, Sensitivity analysis= not significant.

Figure 2. Forest plot of included studies investigating the effect of Fluoxetine on mortality of COVID-19 patients. I^2^ =72.59, Eager’s test (P value) = could not be calculated, Sensitivity analysis= not significant.

Figure 3. Forest plot of included studies investigating the effect of Fluoxetine and/or Fluvoxamine on mortality of COVID-19 patients. I^2^ =27.09, Eager’s test (P value) = 0.055, Sensitivity analysis= not significant.
